# A systematic review of the international evidence on the effectiveness of COVID-19 mitigation measures in communal rough sleeping accommodation

**DOI:** 10.1093/pubmed/fdad114

**Published:** 2023-07-20

**Authors:** Steven Haworth, Owen Cranshaw, Mark Xerri, Jez Stannard, Rachel Clark, Emma Pacey, Gill Leng, Ines Campos-Matos

**Affiliations:** Addictions and Inclusion Directorate, Office for Health Improvement and Disparities, London SW1H 0EU, UK; Institute for Social and Economic Research, University of Essex, Essex CO4 3SQ, UK; Institute for Social and Economic Research, University of Essex, Essex CO4 3SQ, UK; Addictions and Inclusion Directorate, Office for Health Improvement and Disparities, London SW1H 0EU, UK; Addictions and Inclusion Directorate, Office for Health Improvement and Disparities, London SW1H 0EU, UK; Policy, Systems and Innovations Directorate, Office for Health Improvement and Disparities, London SW1H 0EU, UK; Addictions and Inclusion Directorate, Office for Health Improvement and Disparities, London SW1H 0EU, UK; Addictions and Inclusion Directorate, Office for Health Improvement and Disparities, London SW1H 0EU, UK; Addictions and Inclusion Directorate, Office for Health Improvement and Disparities, London SW1H 0EU, UK

**Keywords:** COVID-19, health policy, homelessness, infection control, systematic review

## Abstract

**Background:**

Accommodations with shared washing facilities increase the risks of severe acute respiratory syndrome coronavirus 2 (SARS-CoV-2) infection for people experiencing rough sleeping and evidence on what interventions are effective in reducing these risks needs to be understood.

**Methods:**

Systematic review, search date 6 December 2022 with methods published *a priori*. Electronic searches were conducted in MEDLINE, PubMed, Cochrane Library, CINAHL and the World Health Organization (WHO) COVID-19 Database and supplemented with grey literature searches, hand searches of reference lists and publication lists of known experts. Observational, interventional and modelling studies were included; screening, data extraction and risk of bias assessment were done in duplicate and narrative analyses were conducted.

**Results:**

Fourteen studies from five countries (USA, England, France, Singapore and Canada) were included. Ten studies were surveillance reports, one was an uncontrolled pilot intervention, and three were modelling studies. Only two studies were longitudinal. All studies described the effectiveness of different individual or packages of mitigation measures.

**Conclusions:**

Despite a weak evidence base, the research suggests that combined mitigation measures can help to reduce SARS-CoV-2 transmission but are unlikely to prevent outbreaks entirely. Evidence suggests that community prevalence may modify the effectiveness of mitigation measures. More longitudinal research is needed.

**Systematic review registration:**

PROSPERO CRD42021292803.

## Introduction

People are defined as sleeping rough if they sleep outside or without adequate shelter.^1^ A higher prevalence of comorbidities,^2^ and lower vaccination rates than the general population,^3–7^ make people experiencing rough sleeping more vulnerable to severe acute respiratory syndrome coronavirus 2 (SARS-CoV-2) infections, coronavirus disease 2019 (COVID-19) complications and other nonrespiratory infections such as tuberculosis.^8^ In March 2020, the ‘everyone in initiative’ was launched to help people experiencing rough sleeping in England to comply with COVID-19 regulations.^9^ To this aim, additional funding was made available for accommodation providers to provide more single occupancy accommodation or to restructure existing accommodations to make them COVID-19-safe (i.e. allow isolation).

To help people experiencing rough sleeping to better adhere to personal protective measures, the UK government maintains that single-occupancy accommodation should be provided wherever possible.^10^

However, in some situations, the need for temporary accommodation for people experiencing rough sleeping may exceed single occupancy availability, and communal facilities may still be needed.^10^^,^^11^

Many of the communal accommodations for people who sleep rough in England typically have shared washing facilities, whilst other aspects such as kitchen use or sleeping arrangements may vary.^11^ Thus, ‘communal accommodation’ for people experiencing rough sleeping within England can be defined as accommodations that have shared washing facilities (e.g. bathrooms).

Communal accommodation increases the risk of COVID-19 outbreaks,^12^^,^^13^ which are defined as two or more test-confirmed cases associated with a specific context within 14 days.^14^ Hence, when communal accommodation is the only option, promotion of vaccinations, improved ventilation, mask-wearing, limiting close contact and frequent hand washing are recommended by the UK government.^10^ However, during the pandemic, most communal accommodations in England were closed;^15^ therefore, the effectiveness of these measures in communal accommodations is unclear. Understanding the efficacy of mitigation strategies in this setting is essential for effective future planning and implementation of guidance and policies intending to protect people experiencing rough sleeping against COVID-19 and other infections. This paper thus systematically reviews the international evidence on the effectiveness of measures to prevent the spread of SARS-CoV-2 infection in accommodations for people experiencing rough sleeping that have shared washing facilities.

## Methods

This systematic review followed the Preferred Reporting Items for Systematic Reviews and Meta-Analyses (PRISMA) guidelines^16^ and was registered with PROSPERO. Review protocol registration number: CRD42021270053.

### Search strategy, selection criteria and screening process

Five electronic databases: MEDLINE, PubMed, Cochrane Library, CINAHL and the WHO COVID-19 Database were searched from database establishment to the 6 December 2022. By combining ‘COVID-19’, ‘transmission’ and ‘setting or population’ terms, searches were restricted to research that investigated SARS-CoV-2 transmission mitigation strategies in communal accommodations for people experiencing rough sleeping.

All study designs that quantitatively assessed the effectiveness of measures to protect against SARS-CoV-2 infections and COVID-19 complications in people experiencing rough sleeping in communal accommodation were included. Shared washing facilities are a common feature of ‘communal accommodation’ for people experiencing rough sleeping in England.^13^ Thus, only studies that evaluated mitigation measures in accommodations that met this definition were included. Although the structure of ‘communal accommodation’ may differ across countries this review aimed to evaluate the effectiveness of mitigation measures in settings similar to the provisions in England. Consequently, when evaluating the effectiveness of interventions in communal accommodation for people experiencing rough sleeping all other structural characteristics (e.g. sleeping arrangements) were considered as mitigation measures. We included modelling studies, surveillance reports, pilot studies and randomized control trials (RCTs), and excluded media articles, reviews and opinion papers. A detailed description of inclusion criteria, search strategies and terms is available in the registered PROSPERO protocol. The final search strategy did not deviate from the protocol.

Database searching was accompanied by grey literature searches and hand searching of reference lists of identified studies and publication lists of known experts. Lead researchers of ongoing relevant RCTs were contacted for potential preliminary reports or findings by the principal investigator. Title, abstract and full-text screening of all identified records were conducted independently and in duplicate by two researchers with almost perfect agreement (95%; Cohen’s *k* = 0.88) and discrepancies were resolved through discussion.

### Risk of bias assessment and data extraction

Two researchers independently assessed the risk of bias for each study using appraisal tools from the Joanna Briggs Institute (JBI).^17^ Two reviewers independently and in duplicate rated each domain and computed an overall risk of bias rating for each study (low, moderate and high). Discrepancies were resolved through discussions with consensuses reached on all records. Due to the anticipated low number of available literature in this review, no predefined exclusion quality cut-off was used.

Using a pre-defined and previously used^12^ Excel spreadsheet, the following information was extracted for all retained records: the first author with publication year, study design, study setting/country, target population/sample, mitigation measures assessed, outcomes measured including date measured and method used and main findings. Data extraction was completed independently and in duplicate by two researchers and subsequently combined, and discrepancies were independently checked by a third researcher.

### Data analysis and synthesis

Due to the heterogeneity in the mitigation strategies assessed, the conceptualization of mitigation approaches and how the outcomes were reported across studies, meta-analysis was not possible here and a narrative thematic analysis was conducted. The results were organized by intervention type.

## Results

### Study characteristics

**Fig. 1:**
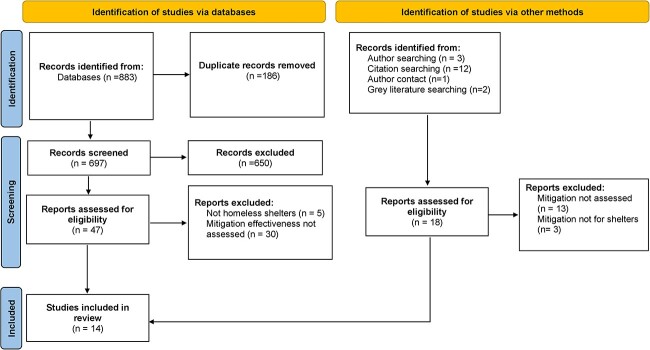
PRISMA search flow diagram.

We identified 883 records, including 186 duplicates through database searching. 697 unique abstracts were screened and assessed for eligibility by two researchers in duplicate, of which 650 were excluded. The remaining 47 records and the 18 identified from other sources (65 in total) were sought for full-text screening. 51 articles were excluded at this stage; 43 for not assessing the effectiveness of mitigation measures and another 8 because they did not meet our definition of communal accommodation for people experiencing rough sleeping.^18–25^ The remaining 14 articles were included in this review (Fig. 1).

Studies were conducted in the United States (USA), England, France, Singapore and Canada. Two studies^26^^,^^27^ were longitudinal. Ten studies were surveillance reports,^20^^,^^26–34^ one was a pilot intervention report^35^ three were modelling or simulation studies.^36–38^ All studies began their surveillance between March and April 2020, capturing the peak of the first wave of the COVID-19 pandemic and before vaccine rollouts in their respective countries.^39–42^ Homelessness was defined inconsistently across the included studies. Nine studies included samples that contained only staff and/or residents within shelters, hostels and hotels used as emergency accommodations for people experiencing homelessness. The other five studies included additional accommodation types (e.g. squats) and/or groups of people that are living in precarious conditions (e.g. migrant workers). See Table 1, for a summary of included studies.

**Table 1:** Characteristics of synthesized studies

Characteristic	Number of studies
Study design	
Cross-sectional	12
Longitudinal	2
Study type	
Modelling/Simulation	3
Pilot intervention	1
Surveillance report	10
Country of study	
United State of America (USA)	6
England	1
France	4
Singapore	1
Canada	2
Outcomes^*^	
Infection incidence/transmission risk or rate	13
Number of and severity of outbreaks	2
Hospital admissions/bed use	3
The severity of infections/death	1
Population	
Staff/residents in shelters/hostels/hotels for homeless people	9
Includes other accommodation and/or sub-groups of people	5

^*^ For studies that reported on multiple outcomes, each of the outcomes was tabulated separately in this table.

### Risk of bias

The risk of bias was rated as low in three studies,^26^^,^^36^^,^^38^ moderate in eight studies,^20^^,^^27^^,^^29–31^^,^^34^^,^^35^^,^^37^ and high in three studies.^28^^,^^32^^,^^33^ The risk of bias for each study is presented in Tables 2 and 3.

**Table 2:** Summary of synthesized surveillance studies

Reference, Country	Study design, and duration	Mitigation measure(s)	Sample	Outcome measure (s)	Summary of findings	Risk of bias
O’Shea *et al*., 2021^35^ *Hamilton, Canada*	Cross-sectional uncontrolled pilot intervention 17 March –30 April 2020	Opening of surge shelters, on-site rapid symptomatic testing, and available isolation space	8 Shelters: 104 residents, and 141 staff	Incidence	1% of residents and 5% of staff tested positive for COVID-19 compared with an estimated prevalence of 5–7% in the local community	Moderate
Tan and Chua, 2020^28^ *Singapore (Nationwide)*	Cross-sectional surveillance report March 2020	Increased bed spacing (1 metre apart). Opening of surge shelters, and staggered meal/shower times	35 shelters: serving 700 residents	Reported Outbreaks	No known outbreaks were reported in homeless shelters in March 2020	High
Husain *et al*., 2021^29^ *Paris, France*	Cross-sectional surveillance report 1 March–31 May 2020	Sleeping arrangements	Residents from 3 shelters	Hospitalization risk, and incidence	The estimated risk of hospitalization did not differ with the number of residents sharing a room. The drop-in day centre had higher infection rates than the healthcare centres (90.6% versus 63.2%, *P* = 0.0042)	Moderate
Chang *et al*., 2022^27^ *Chicago, USA*	Longitudinal surveillance report 30 March –6 May 2020	Cleaning, Hand sanitizer, universal mask-wearing, daily temperature and symptom screening, on-site isolation, off-site isolation, lockdown on-site, and universal PCR-testing	1 shelter: 445 residents and staff	Incidence	The inclusion of increased hygiene, frequent site cleaning, universal mask-wearing, three times weekly PCR testing, and off-site isolation reduced new cases from 45% on the 8 April 2020 to 11% in 16 April and 28 April 2020 and 1% in May 2020	Moderate
Loubiere *et al.*, 2021^30^ *Marseille, France*	Cross-sectional surveillance report 1 June–5 August 2020	Sleeping arrangements, and resident transience	48 rough sleeping locations: 1156 people experiencing rough sleeping	Incidence	Private rooms and shared rooms showed a similar incidence (5.31% versus 5.67%, respectively, *P* = 0.896), less than a month in emergency shelters was associated with a lower incidence (3.61% versus 10.57%, *P* < 0.001) and changing accommodation showed no change (7.01% versus 4.52, *P* = 0.08)	Moderate
Roederer *et al*., 2021^31^ *Paris and Seine-Saint-Denis, France*	Cross-sectional surveillance report 23 June–2 July 2020	Composite close contacts score containing: sleeping arrangements, and bathroom and kitchen sharing	10 emergency shelters and hotels, 1 food distribution site, and 1 migrant worker dormitory: 818 (124 migrant workers).	Incidence	Reducing the amount of time that residents spent in close contact with others (15 minutes or more within 1 metre) was associated with lower odds of being seropositive (Medium: OR = 2.7 (1.5–5.1), High: OR = 3.4 (1.7–6.9)	Moderate
Ly *et al.*, 2021^26^ *Marseille, France*	Longitudinal surveillance report 26 March–5 November 2020	Increase to 24-hour operation, relocation of some residents to hotels, universal mask-wearing, frequent handwashing, and improved coughing etiquette	4 shelters (only 3 had follow-ups): 646 residents 411 homeless, 77 asylum seekers, 58 people living in precarious conditions) and 152 staff	Incidence	Following the implementation of mitigation measures all three shelters that received follow-up assessments showed lower incidence (down to 1.1% from 20.6% by the end of the study). Mitigation measures helped to keep infection rates lower during the second wave in France (7.1% compared with 20.6% in the first wave	Low
Karb *et al*., 2020^32^ *Rhode Island, USA*	Cross-sectional surveillance report 19 April–24 April 2020	Increased bed spacing (6 feet apart), daily symptom screening, universal mask-wearing, daily updates and resident transience	5 shelters: 229 residents	Incidence	Shelters that prohibited new residents showed no cases of COVID-19. Other shelters that allowed new residents showed that residents that stayed for 14 days or more in the same shelter were less likely to contract COVID-19.	High
Rogers *et al*., 2021^33^ *Washington, USA*	Cross-sectional surveillance report 1 Jan–24 April 2020	Sleeping arrangements	14 shelters: 1434 encounters/tests	Incidence	Higher positivity rates were found in residents that slept in a communal room or space (2.3%) compared with those that slept in private spaces (1.38%). 86% of all identified cases were in participants who slept in a communal space in the past week (versus 14% in private rooms)	High
Self *et al*., 2021^34^ *USA (7 states)*	Cross-sectional surveillance report 30 March –1 June 2020	21 Individual mitigation measures (some shelters may implement multiple simultaneously)	63 shelters: Median number of residents per shelter is 63 (range = 7–364)	Prevalence: High (>2.9%) Very high (>10%)	Shelters that positioned beds in a head-to-toe sleeping position and excluding symptomatic staff from working were less likely to have a high infection prevalence, and Shelters that provided medical services available were less likely to have a very high infection prevalence.	Moderate
Kiran *et al*., 2021^20^ *Toronto, Canada*	Repeated cross-sectional surveillance report 1 April–31 July 2020	Resident transience	20 shelters: 872 residents	Incidence	Shelter residents who tested positive were less likely to have visited another shelter in the last 14 days (0% versus 18%, *P* < 0.01).	Moderate

**Table 3:** Summary of synthesized modelling studies

Reference, Country	Mitigation measure(s)	Calibration data	Outcome measure(s)	Simulation effect estimates	Risk of bias
Baggett *et al*., 2020^36^ *Boston, USA*	7 different combinations of daily symptom screening, PCR testing for symptomatic residents and staff, Universal PCR testing, and isolation in other accommodations	Data from 2019 that included 2258 residents from homeless shelters	Incidence, hospital bed use, and total days in hospital	Universal PCR testing every 2 weeks and isolation in an alternative care site was found to be the most effective and cost effective strategy: ***R_0_ = 2.6:*** 14% fewer infections, 86% lower hospital bed use, and 84% less hospital days ***R_0_ = 1.3:*** 62% fewer infections, 89% lower hospital bed use, and 96% less hospital days ***R_0_ = 0.9:*** 46% fewer infections, 80% lower hospital bed use, and 90% less hospital days	Low
Lewer *et al*., 2020^37^ *England* *(Nationwide)*	Private bedroom and washroom facilities, isolation in other accommodations, lockdown in accommodation, closure of communal spaces, hand hygiene promotion, and physical distancing	Data from March 2020 that included 46 565 residents experiencing homelessness (35 817 living in 1065 hostels for homeless people, 3616 sleeping in 143 night shelters, and 7132 sleeping outside)	Incidence, hospital admissions, deaths, and ICU admissions	**Wave 1:** 21 092 fewer cases (45.3% reduced cumulative incidence), 92% fewer hospital admissions, 92% fewer deaths, and 91% fewer ICU admissions. **Wave 2:** 11 168 fewer cases (24.0% reduced cumulative incidence), 89% fewer hospital admissions. 89% fewer deaths, and 89% fewer ICU admissions.	Moderate
Chapman *et al*., 2021^38^ *Boston, San Francisco, and Seattle, USA*	Daily symptom screening, twice-weekly PCR, universal masking, and relocation of some residents to hotels	Data from PCR testing during 28 March–10 April 2020 conducted in 5 Shelters (1 in San Francisco, 1 in Boston, and 3 in Seattle) on 788 residents and 137 staff.	Incidence, and probability of averting an outbreak	**Incidence reduction:** Community incidence = 0: R_0_ = 1.5 = 96%; R_0_ = 2.9 = 97%; R_0_ = 3.9 = 98%; R_0_= 6.2 = 92% Community incidence = 122/1 M/day: R_0_ = 1.5 = 72%; R_0_ = 2.9 = 90%; R_0_ = 3.9 = 92%; R_0_ = 6.2 = 88% Community incidence = 260/1 M/day: R_0_= 1.5 = 66%; R_0_ = 2.9 = 86%; R_0_ = 3.9 = 90%; R_0_= 6.2 = 79% Community incidence = 439/1 M/day: R_0_= 1.5 = 63%; R_0_ = 2.9 = 84%; R_0_= 3.9 = 86%; R_0_= 6.2 = 75% **Probability of averting an outbreak:** ***R_0_* = 1.5:** OR = 0.74 (95% CI = 0.40–1.00) ***R_0_* = 2.9:** OR = 0.42 (95% CI = 0.13–0.99) ***R_0_* = 3.9**: OR = 0.29 (95% CI = 0.07–0.97) ***R_0_* = 6.2:** OR = 0.19 (95% CI = 0.02–0.91)	Low

Bolded and italicized values refer to the SARS-CoV-2 Reproduction number (R_o_).

### Study findings

#### Individual interventions

Eight cross-sectional studies report the effectiveness of individual interventions, including single occupancy rooms, resident mobility, physical distancing between residents and exclusion of symptomatic staff.^20^^,^^28–34^ Despite reporting the efficacy of each intervention independently, not all mitigation measures were implemented in isolation (see Table 2 for more details).

The effectiveness of sleeping in single occupancy rooms and restricting resident mobility is inconsistent. Two reports from France found that sleeping in communal rooms was not associated with a higher SARS-CoV-2 infection rate or risk of hospitalization compared with sleeping in a private room,^29^^,^^30^ whereas in the USA sleeping in communal rooms was associated with higher rates of positivity compared with those sleeping in a private room.^33^ Similarly, the effects of resident mobility varied. In a surveillance report from Canada, residents who remained in the same accommodation for 14 days were less likely to test positive.^20^ Conversely, in a report from France changing accommodations showed no relationship with odds of being seropositive compared with not changing accommodations.^30^ In France, spending less than a month in emergency shelters was associated with lower odds of being seropositive (compared with more than a month).^30^ In a report of six US shelters, despite implementing multiple other infection control practices (see Table 2 for details) only accommodations that prohibited new residents reported no outbreaks.^32^

Measures reducing contact between residents also showed conflicting results. A report in France found that limiting the frequency that residents spent more than 15 min within 1 m of other residents and staff was associated with lower odds of being seropositive.^31^ The study reduced close contact by reducing the number of residents sharing sleeping, cooking and washing facilities. Compared with having 5 or fewer close contacts, more close contacts were associated with greater odds of testing positive for SARS-CoV-2 (6–9 close contacts: OR = 2.7, 95% CI = 1.5–5.1 and >10 close contacts: OR = 3.4, 95% CI = 1.7–6.9). This is consistent with a report from Singapore that suggested there were no reported outbreaks in homeless shelters that had increased bed spacing and staggered meal and shower times.^28^ However, because no further information was available in these studies it is unclear precisely how physical distancing was defined in these contexts. On the other hand, a cross-sectional surveillance report of sixty-three shelters across seven states in the USA found that increasing bed spacing to 3 feet apart and filling fewer than 74% of beds was not associated with lower SARS-CoV-2 prevalence.^34^ Instead, positioning beds head-to-toe and excluding symptomatic staff from working was associated with reduced odds of reporting SARS-CoV-2 prevalence above 2.9% (the median of the 7-day average of the six counties in the study). However, it is not clear whether these shelters implemented head-to-toe sleeping and/or excluded symptomatic staff from working individually or simultaneously with other measures.

#### Combined measures

A cross-sectional uncontrolled pilot study^35^ and two longitudinal studies^26^^,^^27^ looked at a combination of measures to reduce SARS-CoV-2 transmission risk.

The strongest available evidence comes from a longitudinal report (with a low risk of bias) of four shelters (and associated hotels used for depopulation) in France that were assessed from March to November 2020.^26^ In this report, reducing the density of communal accommodation, encouraging good hygiene practices, and increasing social distancing helped to reduce the SARS-CoV-2 infection rate and helped to keep infection rates lower during subsequent waves of SARS-CoV-2, but did not prevent infections entirely. The study reported a decline from 21% of infected residents during the first wave of SARS-CoV-2 (March 2020) to 7% in the middle of the second wave (September 2020), following the implementation of the suggested mitigation measures.^26^ Similarly, a report from the USA revealed that increased accommodation cleaning, more frequent hand washing, universal mask-wearing, off-site isolation, daily symptom screening, PCR testing three times per week, and prohibiting residents who left the accommodation to return was found to reduce the SARS-CoV-2 infection rate from 45% of infected residents in April 2020 to less than 1% in May 2020.^27^

A Canadian uncontrolled pilot study^35^ suggests that by reducing shelter density, increasing social distancing, providing rapid on-site testing, universal mask-wearing and providing an isolation area for people awaiting test results the researchers were able to keep shelter SARS-CoV-2 infection rates at 1%, which was below that of the general population (estimated 5–7%), but could not entirely stop new infections within the shelter.

#### Modelling studies

Three modelling studies were selected: two simulating shelters in the USA (both rated as having a low risk of bias)^36^^,^^38^ and in England^37^ (summarized in Table 3).

Both US studies estimated that daily symptom screening, relocation of some residents to hotels (to reduce shelter density), universal mask-wearing, off-site isolation and twice weekly PCR testing could help to reduce SARS-CoV-2 infections by between 62%^36^ and 96%^38^ when the reproduction number (R_0_) is low (R_0_ = 1.3 and 1.5, respectively). Under a low R_0_, it was estimated that together these mitigation measures had a 74% chance to avert an outbreak in the communal accommodation; however, as the community prevalence increases (to 2.9, 3.9 and 6.2), the effectiveness of these mitigation measures to prevent an outbreak declined to 42%, 29% and 19%, respectively.^38^ The simulation in England also estimated that combined packages of interventions could help dramatically reduce infection rates by 45% and hospitalizations by 92% during the first wave of COVID-19 in the country, but during the second spike in community prevalence, whilst the measures were still in place the estimated effectiveness dropped to a reduction of 24% in SARS-CoV-2 infections and 89% fewer hospitalizations.

## Discussion

### Main findings

Existing evidence on the effectiveness of interventions to reduce SARS-CoV-2 transmission and COVID-19 complications among people experiencing homelessness in communal accommodation is weak, due to a reliance on cross-sectional study design and modelling studies as well as the risk of bias in study methodologies. However, the evidence shows that the implementation of multiple mitigation measures together can help reduce SARS-CoV-2 infections in communal accommodations, although not enough to stop all outbreaks. The pilot intervention in Canada^35^ and the longitudinal surveillance report in France^26^ provided the strongest evidence upon which to assess mitigation measures in this setting. Yet, many of the other studies lacked the critical information required to understand and assess the implemented interventions. Continued and better quality research into how to mitigate COVID-19 and other diseases in communal accommodation is needed, particularly taking into account how factors such as the prevalence of SARS-CoV-2 in the community can influence the effectiveness of mitigation measures.

### What is already known

Communal accommodation is well recognized to increase the risk of transmission of SARS-CoV-2^12^ and could accelerate the spread of other airborne pathogens, such as TB.^8^ Severe complications from COVID-19 and TB are far more pronounced in vulnerable populations that have increased comorbidities and are under-vaccinated, such as people experiencing rough sleeping, migrant workers and refugees and asylum seekers.^2^^,^^43^^,^^44^ Differing physical, social, economic and environmental factors increase the susceptibility to hazards for each of these populations,^45^ which are further exacerbated by poor living conditions.^46^ Thus, because it is precisely these populations that often reside in precarious housing or are living in overcrowded or communal accommodations (e.g. migrant processing centres, night shelters),^47^ understanding how to protect these vulnerable populations from life-threatening diseases in communal accommodations is crucial.

### What this study adds

This review adds to the previous literature by compiling the available international evidence to assess the effectiveness of COVID-19 mitigation strategies in communal accommodations for people experiencing rough sleeping. The findings from this review suggest that implementing multiple mitigation measures simultaneously, such as early identification and isolation of positive cases, reducing accommodation density, reducing close contacts and promoting better hygiene and mask-wearing, could under some circumstances help reduce SARS-CoV-2 transmission in communal settings. Similar mitigation measures have been shown to help reduce the spread of SARS-CoV-2 in schools^48^ and shelters for asylum seekers^49^ and other airborne transmissible conditions, like TB^50^ and influenza.^51^

However, this review also exposes the weakness of the available evidence concerning assessing the effectiveness of COVID-19 mitigation measures in communal accommodations for people experiencing rough sleeping. The literature is made up of mostly cross-sectional studies that were conducted during the first wave only and before vaccine rollouts in their respective locations. Because vaccine uptake is lower in people experiencing rough sleeping^6^ and some evidence suggests that there is still transmission in communal settings following vaccination,^52^ it is increasingly important to understand what interventions are effective at reducing transmission risks in communal accommodations for people experiencing rough sleeping. However, with most of the available evidence being cross-sectional and many with a high risk of bias, more high-quality research that allows causality to be determined is needed to help in identifying the measures that are the most effective.

Furthermore, despite the recognition that good ventilation is likely to play a role in protecting people in communal accommodation against SARS-CoV-2, TB and Influenza,^10^^,^^50^^,^^51^ no studies captured in this review assess ventilation as a mitigation measure in communal accommodation for people experiencing rough sleeping.

Finally, this review demonstrates that factors such as community prevalence can influence how effective different mitigation measures are. For instance, a modelling study estimated that universal mask-wearing on its own would reduce the infection rate in the shelter by 86% when the community prevalence was low, but only by 56% when community infection rates were high. This is important because during periods of low community prevalence individual mitigation measures may be sufficiently effective. France and Canada’s national lockdown was stricter than in the USA,^53^ where large social and religious gatherings still occurred.^54^ Thus, national policies and behaviours and attitudes of residents in the surrounding communities may also influence the effectiveness of mitigation measures.

### Limitations of study

There are, however, some caveats of this review that need to be considered. To begin with, this review did not include pre-print servers which may have resulted in some available evidence being missed. Additionally, the study designs of the literature captured by this review are too limited to allow concrete recommendations on individual mitigation measures for policymakers and accommodation providers to be provided. Finally, variability in the types of communal accommodations reported on and the country-level differences in the COVID-19 landscapes in the captured studies make relating the findings from this review to the UK setting more difficult.

## Conclusions

This review reveals that the available evidence to assess the effectiveness of COVID-19 mitigation strategies in communal accommodations for people experiencing rough sleeping is weak. Yet, together it suggests that even though no intervention or ‘package of interventions’ is likely to prevent outbreaks, they can be used to reduce SARS-CoV-2 infections and COVID-19 complications in this setting. Combining the opening of additional accommodations to reduce the density in communal shelters, universal mask-wearing and proper hygiene practices (i.e. hand washing, less face touching, and good coughing etiquette) may help reduce infection rates in communal accommodations for people experiencing rough sleeping. However, the evidence also suggests that situational factors such as community prevalence will play a role in the efficacy of implemented mitigation packages. It is unclear whether other individual or combinations of mitigation strategies not assessed here could prevent outbreaks or further reduce infection risks in communal accommodations for people experiencing rough sleeping. Thus, better quality research is urgently needed in this area.

## Conflict of interest

There are no conflicts of interest to declare.

## Funding

This research did not receive any specific grant from funding agencies in the public, commercial or not-for-profit sectors.

## Authors’ contributions

SH contributed to the design of this research and participated in literature searching, data extraction, data synthesis/analysis, manuscript preparation, and reviewing and editing. OC participated in literature searching, data extraction, data synthesis/analysis, manuscript preparation, and reviewing and editing. MX and JS contributed to the design of the research and manuscript preparation and editing. RC, EP, and JL participated in research design and manuscript editing. ICM was responsible for the supervision of the entire project and participated in the design of the research and reviewing and editing of the manuscript.

## Notes

**Steven Haworth,** Research Officer

**Owen Cranshaw,** Research Assistant

**Mark Xerri,** Programme Manager: Rough Sleeping Drug & Alcohol Treatment

**Jez Stannard,** Senior Programme Manager: Rough Sleeping Drug & Alcohol Treatment

**Rachel Clark,** Head of Evidence and Evaluation

**Emma Pacey,** Policy Lead for Inclusion Health

**Gill Leng,** National Health and Homelessness Adviser

**Ines Campos-Matos,** Deputy Director for Inclusion Health
